# Clinical effect of minimally invasive technique of internal fixation device removal in patients with healed long tubular bone fractures

**DOI:** 10.12669/pjms.39.1.6967

**Published:** 2023

**Authors:** Chaoyi Li, Wei Lin, Guozhi Wu, Linkang Zhang, Pijun Zhang

**Affiliations:** 1 Chaoyi Li, Department of Joint Surgery, The Second Affiliated Hospital of Hainan Medical College, Haikou 570311, Hainan Province, P.R. China; 2 Wei Lin, Department of Joint Surgery, The Second Affiliated Hospital of Hainan Medical College, Haikou 570311, Hainan Province, P.R. China; 3 Guozhi Wu, Department of Joint Surgery, The Second Affiliated Hospital of Hainan Medical College, Haikou 570311, Hainan Province, P.R. China; 4 Linkang Zhang, Department of Clinical Medicine, Hainan Medical College, Haikou 571199, Hainan Province, P.R. China; 5 Pijun Zhang, Department of Joint Surgery, The Second Affiliated Hospital of Hainan Medical College, Haikou 570311, Hainan Province, P.R. China

**Keywords:** Minimally Invasive Techniques, Long Tubular Bone Fractures, Internal Fixation Devices

## Abstract

**Objective:**

To investigate the clinical effect of minimally invasive technique applied to internal fixation removal in patients with healed long tubular bone fractures.

**Methods:**

The records of patients with internal fixation of long tubular bone fracture who underwent the removal of the internal fixation device after fracture healing in The Second Affiliated Hospital of Hainan Medical College from May 2020 to December 2021 were reviewed. According to the different operation methods of taking out the internal fixation device, patients were divided into minimally invasive group (n=40) and traditional group (n=45). The perioperative indexes, levels of inflammatory factors tumor necrosis factor-α (TNF-α), C-reactive protein (CRP), Karnofsky Performance *Score* (KPS), visual analog scale (VAS) pain score and complications were compared between the two groups.

**Results:**

The drainage volume, bleeding volume, incision length and hospital stay in the minimally invasive group were significantly lower than those in the traditional group (*P<*0.05). The KPS score of minimally invasive group was significantly higher than that of traditional group at one week and one month after the operation, and the VAS score of minimally invasive group was significantly lower than that of traditional group at one day and one week after the operation (*P<*0.05). The levels of TNF-α and CRP in the observation group were significantly lower than those in the control group(*P*<0.05). There was one case of infection in the minimally invasive group, one case of secondary fracture and two cases of infection in the traditional group(*P*>0.05).

**Conclusions:**

Minimally invasive surgery for the removal of the internal fixation device in patients with healed long tubular bone fractures with internal fixation is associated with significantly improved clinical effect, relieved symptoms, reduced inflammatory response, and improved functional recovery of patients.

## INTRODUCTION

Open fractures of long tubular bones are relatively common, and are mainly caused by traffic accidents, falls from heights, and types of trauma, which seriously affect the patient’s limb movement ability.^1^,^2^ Internal fixation devices are often used to treat long tubular bone fractures.^3^ However, when the fracture is healed, the internal fixation device, as a foreign body, may cause stress-induced bone atrophy. Therefore, a second operation is required to remove it.^4^

In cases of long bone fractures, traditional surgery is not only associated with large wound surface, long operation and hospitalization times, high rate of complications, and slow recovery, but also with significant post-surgical pain that seriously impacts patients’ quality of life.^5^ With the development of electronic electro thermal optics and other equipment and technologies, minimally invasive surgery has become a first choice for clinical treatment of long tubular bone fractions, since they are able to minimize the tissue damage and are associated with less bleeding, less postoperative pain, faster recovery, and little or no scars.^6^ The objective of this retrospective study was to investigate the clinical effect of minimally invasive technique used for internal fixation removal in patients with healed long tubular bone fractures, and to assess its impact on functional recovery of the patients.

## METHODS

Medical records of 85 patients (57 males and 28 females) who underwent internal fixation of long tubular bone fractures and removal of internal fixation devices after healing in the second Affiliated Hospital of Hainan Medical College from May 2020 to December 2021 were reviewed. The age of the patients ranged from 22 to 74 years, with an average of (51.59±13.49) years. According to the different operation methods of taking out the internal fixation device, 40 underwent minimally invasive surgery and were set as a minimally invasive group, and 45 patients underwent traditional surgery and were set as a traditional group.

### Inclusion criteria:

- X-ray images showing bony healing of all fractures
- Successful removal of the internal fixation device
- Complete patient data
- Postoperative follow-up records available for more than six months.

### Exclusion criteria:

- Patients with malignant tumor
- Patients with severe intestinal, liver, kidney and cardiopulmonary diseases
- Patients with severe cardiovascular disease and coagulation disease
- Congenital malformation of long tubular bone

### Ethical Approval:

The Ethics Committee of our hospital approved this study. Ref.LW2022024 dated February 16^th^ 2022.

Appropriate anesthesia methods were selected based on the specific indications local infiltration anesthesia was used for clavicle, fibula and ulna brachial plexus block anesthesia was used for humerus and radius intraspinal anesthesia was used for femur and tibia and general anesthesia with endotracheal intubation was used for the repair of multiple fractures.

Traditional surgery was performed by making an incision was made according to the original scar. Tissues were peeled layer by layer, exposing the fixed steel plate and screw tail of the original operation. Built-in and nail tail groove were removed with an electric knife together with the surrounding scar tissue, and bleeding was stopped. Screws were unscrewed and taken out in turn, and the excess callus around the steel plate was removed with a bone knife. One end of the steel plate was pried up with a periosteal stripper to expose it outside the skin, one end of the steel plate was clamped with a vise, rotated 180°in the positive and negative directions respectively, and then the internal fixation device was taken out smoothly, and the bleeding stopped completely. After repeated flushing with normal saline, a drainage sheet or a drainage tube was placed, and the bandage was sutured layer by layer for pressure binding.

Minimally invasive surgery was performed as follows: X-ray film was used as a reference before the operation. The specification and model of internal fixation device was measured and recorded, and the spacing of screw and nail tail groove was accurately calculated. Using a small cutter, one end of the removable steel plate was cut, the steel plate and screw tail cap were fully exposed. Appropriate nail removal tools were selected, and the angle and the direction of the probe were determined according to the direction of the steel plate and the distribution of screws. Nail removal working channel was established through the positioning needle, a cut of 3-5 centimeter was made, exposing the nail tail groove that was then unscrewed and screws taken out in turn.

C-arm X-ray machine was used as an auxiliary in difficult cases, and the electric knife was used to remove the proliferative tissue embedded around the screw hole and to stop bleeding completely. One end of the steel plate was lifted with a periosteal stripper and clamped with a vice. The other end was temporarily fixed with a vascular clamp or screw as the center, rotated clockwise and counterclockwise for several times respectively. The steel plate was removed, and the wound rinsed with normal saline. A drainage piece or drainage tube were retained, and bandages were sutured layer by layer for pressure bandage.^7^,^8^

The following indexes were collected for all the patients. 1) Perioperative indexes such as operation time, bleeding volume, drainage volume, incision length and hospital stay. 2) Recovery of patients at one week, one month and three months after operation was evaluated by Karnofsky Performance *Score* (KPS), with a full score of 100. The higher the score, the better the health status of patients and the stronger the ability to endure the pain caused by the treatment. The postoperative pain was evaluated by visual analogue scale (VAS) at one day, one week and one month after the operation. The full score was 10. The higher the score, the more intense the pain was. 3) The indexes of inflammatory factors before the operation, one day and three days after the operation were measured as follows: under fasting condition, four milliliter of peripheral venous blood was collected, centrifuged at the speed of 3000 r/minute, and the serum was separated after 10 minutes. The serum tumor necrosis factor-α(TNF-α) and C-reactive protein (CRP) were measured by enzyme-linked immunosorbent assay (Shanghai kanglang Biotechnology Co., Ltd.) according to the manufacturer’s instructions 4) The incidence of complications within six months after the operation.

### Statistical Analysis:

SPSS 22.0 statistical software was used for statistical analysis. The measurement data were expressed in (*χ̄*±*S*), and independent sample t-test was adopted. The counting data was expressed in n (%), and *χ^2^* test was used. *P*<0.05 indicated statistically significant difference.

## RESULTS

A total of 85 patients were included in this study, 45 in the minimally invasive group and 40 in the traditional group. There was no significant difference in gender, age, fracture location, cause of injury and fracture classification between the two groups (*P*>0.05) (Table-I).

**Table-I:** Comparison of general clinical data between the two group’s n (%),(*χ̅*±*S*).

Index	Classification	Minimally invasive group (n=40)	Traditional group (n=45)	χ^2^/t	P
Gender	Male	25 (62.5)	32 (71.11)	0.711	0.399
	Female	15 (37.5)	13 (28.89)		
Age		52.10±13.88	51.13±13.28	0.328	0.744
Fracture site	Femur	14 (35.0)	21 (46.67)	2.472	0.650
	Tibia	9 (22.5)	6 (13.33)		
	Humerus	5 (12.5)	7 (15.56)		
	Radius	5 (12.5)	6 (13.33)		
	Ulna	7 (17.5)	5 (11.11)		
Cause of injury	Traffic accident	27 (67.5)	28 (62.22)	1.276	0.528
	Personnel conflict	7 (17.5)	6 (13.33)		
	Falling from height	6 (15.0)	11(24.44)		
Fracture classification	Simple fracture	24 (60.0)	24 (53.33)	0.502	0.778
	Wedge fracture	9 (22.5)	13 (28.89)		
	Complex fracture	7 (17.5)	8 (17.78)		

The amount of drainage, bleeding, hospital stay and incision length in the minimally invasive group were significantly lower than those in the traditional group (*P*<0.05), but there was no significant difference in the operation time between the two groups (Table-II).

**Table-II:** Comparison of perioperative related indexes between the two groups (*χ̅*±*S*).

Groups	Operation time (minute)	Drainage volume 24 hours after operation (ML)	Intraoperative bleeding volume (ML)	Length of stay (day)	Wound length (cm)
Minimally invasive group (n=40)	61.42±12.54	44.30±10.93	103.10±24.44	5.02±1.38	5.07±1.18
Traditional group (n=45)	60.22±10.35	72.22±14.51	163.29±36.61	7.93±2.08	12.04±2.23
t	0.484	-9.920	-8.797	-7.653	-18.229
P	0.630	<0.001	<0.001	<0.001	<0.001

At one week and one month after the operation, the KPS score of minimally invasive group was higher than that of traditional group (*P*<0.05), but there was no significant difference between the two groups at three months after the operation. At one day and one week after the operation, the VAS pain score of minimally invasive group was significantly lower than that of traditional group (*P*<0.05), but there was no significant difference between the two groups at one month after the operation (Table-III).

**Table-III:** Comparison of KPS and VAS scores between the two groups after operation (*χ̅*±*S*).

Groups	KPS score			VAS score		

	1^st^ week after operation	1^st^ month after operation	3^rd^ months after operation	1^st^ day after operation	1^st^ week after operation	1^st^ month after operation
Minimally invasive group (n=40)	63.72±5.59	80.07±6.21	88.45±5.83	4.35±0.95	2.60±1.01	1.50±0.55
Traditional group (n=45)	55.71±5.42	74.35±6.50	86.75±4.26	5.73±1.09	3.33±0.88	1.62±0.65
t	6.699	4.132	1.541	-6.186	3.584	-0.927
P	<0.001	<0.001	0.127	<0.001	0.001	0.357

There was no significant difference in the levels of TNF-α and CRP between the two groups before the operation (*P*>0.05). While the levels of TNF-α and CRP in the two groups increased at one and three days after the operation, these levels were significantly higher in the traditional group compared to the minimally invasive group (*P*<0.05) (Table-IV).There was one case of infection in minimally invasive group, one case of secondary fracture and two cases of infection in traditional group. The difference was not statistically significant (*P*>0.05).

**Table-IV:** Comparison of TNF-α and CRP levels in groups (*χ̅*±*S*).

Groups	TNF-α (pg/mL)			CRP (mg/L)		

	Preoperative	1^st^ day after operation	3^rd^ day after operation	Preoperative	1^st^ day after operation	3^rd^ day after operation
Minimally invasive group (n=40)	148.77±41.68	213.20±46.38	170.47±41.11	34.75±11.85	43.30±12.04	36.77±11.79
Traditional group (n=45)	151.98±39.33	238.42±45.97	190.86±49.95	35.91±13.60	52.33±14.20	42.11±8.10
t	-0.364	-2.514	-2.039	-0.417	-3.141	-2.403
P	0.718	0.014	0.045	0.678	0.002	0.019

## DISCUSSION

The results of this study showed that in the removal of internal fixation device after the healing of long tubular bone fracture internal fixation, the use of minimally invasive technology achieved good results. Minimally invasive surgery was associated with significantly better perioperative indexes, improved postoperative recovery of health function and better inflammatory factor levels.^9^

Traditional methods of internal fixation device removal often require a larger surgical incision and stripping more surrounding tissue for better exposure, especially for patients with serious fracture injury. This may lead to excessive scar tissue and poor blood circulation after internal fixation treatment. Reoperation is also associated with the increased risk of surrounding tissue necrosis, incision infection etc.^10^ However, due to the long-term stress, if left unremoved, internal fixation device may cause tissue hyperplasia and callus, and affect the original deformation ratio, which may cause bone atrophy and partial bone nonunion.^11^ Tumor necrosis factor-α (TNF-α) and C-reactive protein (CRP) are typical inflammatory indexes. In this study, TNF-α and CRP in the two groups were significantly higher before the treatment as compared to their levels after the surgery.

We may speculate that the strain forces, due to the unremoved internal fixation devices, caused inflammatory reaction around the steel plate, resulting in the increased inflammatory indexes. After the removal of the internal fixation device, the strain force was eliminated, the fracture site repair was improved, and the inflammatory index decreased significantly. Moreover, postoperative TNF-α and CRP levels were significantly lower in patients that underwent minimally invasive procedure, as compared to patients that underwent a traditional removal surgery, which shows that minimally invasive surgery is better than traditional surgery in reducing inflammatory indexes.^12^

The removal of internal fixation device is often difficult due to a number of factors, such as the mismatch of instruments and equipment, screw fracture, callus wrapping, filament winding, etc.^13^ Liu K et al^14^ pointed out that the application of minimally invasive technology for traditional extraction of the internal fixation devices has the advantages of accurate positioning, small incision, small trauma and fast recovery. Minimally invasive surgery relies on taking out the internal fixation with a small incision using a C-arm X-ray machine for guidance and has the advantages of less bleeding, short incision, pain relief, shorter operation time and improved patient satisfaction.^15^,^16^

Both traditional and minimally invasive methods can effectively remove the internal fixation of fracture in clinical practice, but there are only few related studies on the comparison of their curative effects. Several studies showed that the application of minimally invasive technology for the removal of internal fixation devices minimizes trauma, thus improving fracture healing and the quality of life and functional recovery of patients, in agreement with our results.^17^,^18^

It is important to mention that the removal of internal fixation devices is not limited to two removal methods described in our paper. For the removal of internal fixation for fracture healing, a circular saw can be used to cut off part of the steel plate at the sliding wire screw cap. After taking out the steel plate, the residual steel plate can be clamped with pliers to remove the sliding wire screw smoothly. This method is associated with small trauma and short operation time.^19^ After the removal of the internal fixation device, special attention should be paid to providing adequate postoperative nursing care to prevent complications such as infection and gas gangrene.^20^

### Limitation:

It is a single center study, and only patients with long tubular bone fracture types were included. Additionally, the follow-up time was short. Further multi-center studies with different fracture types and longer follow-up are needed.

## CONCLUSION

Minimally invasive surgery for plate removal after long tubular bone fracture is associated with better clinical effect compared to the traditional surgery. It, can therefore, reduce symptoms and promote faster and better functional recovery of the patients.

### Authors’ contributions:

**CL and WL** conceived and designed the study.

**GW, LZ and PZ** collected the data and performed the analysis.

**CL and WL** were involved in the writing of the manuscript and are responsible for the integrity of the study.

All authors have read and approved the final manuscript.
